# Supplementary material for: Understanding the current acute aortic syndrome (AAS) pathways—The Collaborative Acute Aortic Syndrome Project (CAASP) protocol
Source: PLoS One. 2024 Feb 2;19(2):e0297782. doi: 10.1371/journal.pone.0297782 (PMC10836658; doi:10.1371/journal.pone.0297782)
Supplement: S1 File — (DOCX) [file pone.0297782.s001.docx]

**Supplementary Information**

1. **Radiology Information System (RIS) Search Strategy**

The first steps that must be completed are:

- Register project at local site as a national service evaluation project
- Discuss project with local radiology team for electronic search of cases on radiology information system (RIS).

After completion of these steps, the following RIS search should be used:

- **Time Period to search:** 01/01/2018 to 01/06/2021
- **Modality to search for:** Computed Tomography (CT) Aorta Angiogram
- **Keyword search** within the clinical information section and main body of radiology report section: *‘aortic syndrome’, ‘aortic dissection’, ‘penetrating ulcer’, ‘dissection flap’, ‘intramural haematoma’*

1. **Data Points for Collection**

***Patient demographics***

● Age and Gender

● Ethnicity (White/ Black / Asian / Other) – If other option to write open box text

● Distance from home (if ambulance was called to another location please use this if available) to presenting hospital

● Index of Multiple Deprivation (IMD) Decile

● Aortic Pathology: AD Type A / AD Type B / AD non-A non-B / PAU / IMH

***Presentation Timing Details***

- Time from symptom onset to hospital presentation/ first point of medical contact (hours)
- Mode of ED presentation: direct self-presentation / GP referral / Ambulance / Other (free text)

***Symptoms (all Y/N/ not recorded)***

● chest pain/ sudden onset pain/back pain/ neck pain/ abdominal pain/ migratory pain/ severe intensity pain/ collapse/ focal neurology (e.g. numbness or weakness in limbs)/ other (free text other)

***Patient Risk Factors (all Y/N/ not recorded)***

● Hypertension / Known Aortic Aneurysm / Previous Aortic Dissection / Bicuspid Aortic Valve / History of myocardial infarction / Cardiac Failure / Previous cardiac surgery / Prior catheterization or angioplasty / Known familial aortic disease / Known Marfans or other connective tissue disorder / Diabetes

***Physical Exam (Y/N/not recorded)***

● New murmur on examination / Difference in Bilateral BP (>20 mmHg ) / Pulse deficit (a difference in the apical pulse and the peripheral pulse)/ Vascular Signs (Ischaemic limb?)/ Objective neurological deficit

***Vital Signs/ Presenting clinical state***

● First recorded admission BP (systolic/ diastolic) / HR / RR (in emergency department)

● Hypotension (Defined as BP less than 90 mmHg systolic or 60 mmHg diastolic) (Y/N)

● Presenting with clinical features compatible with circulatory shock (See definition in appendix) (Y/N)

● Presenting with clinical or radiological features compatible with likely cardiac tamponade (See definition in appendix) (Y/N)

● Fever (Defined as >37.5°C) (Y/N)

● Coma / altered consciousness (Y/N)

***Investigations***

- ECG (Normal / Abnormal / Not done)
- With which imaging modality was the diagnosis of AAS first confirmed (Ultrasound incl Echo/CT/MR)
- CT done to confirm diagnosis (Y/N)
- Type of CT study: CTA / CTPA / Non-contrast CT / Portal venous abdomen / Other
- Complicated / uncomplicated disease (Define in supplementary material for data collectors)
- *Time from arrival to hospital to first imaging diagnosis (hours)*
- Was AAD or AAS clinically suspected in the pre-imaging clinical notes? (Y/N) - If No, incidental diagnosis (to be analysed separately)
- Was AAD or AAS mentioned on the imaging request? (Y/N)
- *Time from admission to vascular/ cardiothoracics referral*

***Bloods***:

● Point of care bloods: pH on venous bloods/ blood gas (record value or leave blank if not recorded)

● FBC: Haemoglobin (g/dL)/ WBC (109/L)/ Platelets (109/L) (record value or leave blank if not recorded)

● D-dimer (use absolute cut-off) / Troponin (Normal/ Abnormal/ Not recorded)

● CRP / ALT / Amylase / Lactate / Glucose (record value or leave blank if not recorded)

● Creatinine / eGFR (record value or leave blank if not recorded)

***Treatment***

- Definitive treatment during first admission: Medical (BP control) / Surgery / Endovascular
- Discharge home (Y/N)
- Transfer to another centre (Y/N)
- *Time from CT to the implementation of the documented definitive management plan (Hours) (e.g. if documented definitive management plan is TEVAR then time to TEVAR or time from CT to hospital transfer to aortic centre)*
- Prescribed BP Target (mmHg) – systolic
- Prescribed heart rate target (beats per minute)
- Was an Intravenous antihypertensive/beta blocker used to control blood pressure and heart rate? (Y/N)
- If Yes which was used first line (Tick one):
- Labetalol/ Esmolol/ Metoprolol/ GTN/ Sodium Nitroprusside/ Nicardipine/ Hydralazine/ Verapamil/ Diltiazem/ Other Beta blocker, please specify/ Other calcium channel blocker, please specify/ Other please specify
- If Yes which was used second line (Tick one):
- Labetalol/ Esmolol/ Metoprolol/ GTN/ Sodium Nitroprusside/ Nicardipine/ Hydralazine/ Verapamil/ Diltiazem/ Other Beta blocker, please specify/ Other calcium channel blocker, please specify/ Other please specify/ Second line IV agent not required

● Time from commencement of BP treatment to achieving BP/ HR target (hours)

***Hospital Stay***

- Which of the below best describes the type of ward area to which the patient initially admitted (tick one):
- Intensive care unit (level 3 capability)
- High Dependency Unit (Level 2 capability)
- High observation area (Level 1 capability)
- Coronary Care Unit
- Specialist vascular ward
- None specialist ward
- Admission to a critical care ward (ICU/ HDU/ CCU) during hospital stay (Y/N)
- Length of ICU stay (days)
- Length of total hospital stay (days)
- Alive at discharge (Y/N)
- Alive at 30 days(Y/N)
- Alive at 90 days(Y/N)
- Alive at 6 months (Y/N)
- *COVID status: Negative PCR / Positive PCR / Radiological evidence only/ Clinical concern only for COVID/ not recorded*
